# Hearing impaired persons' experiences with the online Swedish Individualized Active Communication Education (I-ACE) program: A feasibility study

**DOI:** 10.1016/j.invent.2024.100734

**Published:** 2024-03-16

**Authors:** Louise Werther, Elisabet Thorén, Jonas Brännström, Gerhard Andersson, Marie Öberg

**Affiliations:** a Department of Otorhinolaryngology in Östergötland, Department of Biomedical and Clinical Sciences, Linköping University, Linköping, Sweden; b Department of Otorhinolaryngology, Head and Neck Surgery, Audiology Clinic, Skåne University Hospital, Lund, Sweden; c Department of Clinical Science, Logopedics, Phoniatrics and Audiology, Lund University, Lund, Sweden; d Department of Clinical Neuroscience, Karolinska Institute, Stockholm, Sweden; e Department of Behavioural Sciences and Learning, Linköping University, Linköping, Sweden

**Keywords:** I-ACE, eHealth, Hearing loss, Aural rehabilitation, Communication strategies

## Abstract

Even with optimally fitted hearing aids, many individuals with hearing impairment struggle to hear in situations with difficult listening conditions. Active Communication Education (ACE) is an interactive group rehabilitation program aimed at helping people with hearing loss communicate more effectively using communication strategies to better cope with everyday life. To increase accessibility and allow more people to benefit from the ACE program, a modified individualized version was created. The purpose of this study was to examine the feasibility of providing the Swedish Individualized Active Communication Education (I-ACE) program via an online platform and to explore hearing impaired persons' experiences with the program. For five weeks, ten participants completed the Swedish I-ACE through an online platform. The participants were assigned a new chapter to complete each week and later received individual feedback on their work via the platform. The participants were asked to complete an evaluation form regarding the content and their experiences during and after completing the I-ACE. They were later interviewed to provide more detailed information on their experiences with the program. The program completion rate was 80 %. Participants found the I-ACE program to be informative and relevant but somewhat repetitive. However, only a few participants thought of the repetitiveness as negative. Few participants reported difficulties using the platform. This study indicated that it is feasible to provide the I-ACE program via an online platform and that the content of the program is informative, relevant, and comprehensible. Further research evaluating the effects of the I-ACE is warranted.

## Highlights

• Participants found the content of the I-ACE to be informative and relevant.
• Both the I-ACE and the platform used were found to be comprehensible and manageable.
• It is feasible to provide the I-ACE via the examined platform.

## 1 Introduction

Hearing loss is a common chronic condition affecting >20 % of the global population (World Health Organization, 2021). Untreated hearing loss can lead to communication difficulties and increased social isolation, which in turn can adversely affect both psychological and physical health (Boddy et al., 2020). It has also been reported that untreated hearing loss is a potential risk factor for dementia (Livingston et al., 2020). The most common intervention available for hearing loss is audiological rehabilitation through hearing aid fitting. Even with optimally fitted hearing aids, many individuals with hearing impairment still struggle to hear in challenging communication situations that occur in everyday life.

One way to manage communication challenges that remain after hearing aid fitting is through the Active Communication Education (ACE) program. The ACE program is an interactive group rehabilitation program that addresses communication difficulties due to hearing loss and was developed as both an alternative and a supplement to hearing aid fitting (Hickson and Worrall, 2003). The program consists of a series of modules, each addressing common difficult communication situations such as conversations in noisy environments and conversations in group settings. The themes of the different modules are discussed in two-hour weekly sessions for five weeks. The ACE program has been shown to reduce communication limitations and participation restrictions experienced by people with hearing impairment (Hickson et al., 2007; Öberg, 2017; Öberg et al., 2014a).

To make the ACE program available to those who have practical difficulties attending group sessions or who might prefer not to participate in a group setting, the program was modified to create a version that could be completed individually, the Individualized Active Communication Education (I-ACE) program. This individual version similarly showed improved outcomes regarding participation restriction and activity limitations, although these outcomes were not as positive as those reported for the group-delivered ACE program (Hickson et al., 2019). The Swedish version of the I-ACE has been evaluated in two smaller pilot studies where the material was provided via e-mail. These studies showed positive results regarding the emotional and social effects of hearing loss and the use of communication strategies (Annerfeldt and Svensson, 2017; Hagejärd and Teodorescu, 2015).

Previous research has shown that providing audiologic rehabilitation via the internet can be effective at reducing participation restrictions and activity limitations (Brännström et al., 2016; Malmberg et al., 2023; Malmberg et al., 2015; Thoren et al., 2011; Thoren et al., 2014). The use of telehealth in audiology has increased as a result of the COVID-19 pandemic (Eikelboom et al., 2022), and in Sweden 83 % of the population reported having used some form of digital health care service (Internetstiftelsen, 2022). The increased use of telehealth has raised concerns regarding protecting patient privacy and data, urging regulations for safe communication between patients and health care professionals (Garfan et al., 2021). In Sweden, a previous national collaboration between regions resulted in a secure online health portal for the public health care system, 1177.se. Through this portal, residents can access information about healthcare, illnesses and conditions. A section of this portal is called the Support and Treatment platform, where support and/or treatment programs can be assigned to residents. Malmberg et al. (2023) used this platform to provide a support program for hearing aid users, with the majority of participants reporting that the platform was easy to use.

In recent decades, patient and public involvement (PPI) has attracted increased interest in health research and the provision of health care. Positive impacts of PPI, such as improved enrollment in clinical trials and better adherence to treatment and self-efficacy, can be found in all stages of research (Brett et al., 2014; Crocker et al., 2018; Modigh et al., 2021). It is recommended that relevant stakeholders be involved in the development of complex interventions to increase their chances of being effective (O'Cathain et al., 2019). Interventions involving multiple interacting components can be described as complex (Craig et al., 2008). With its different dimensions aiming to improve an individual's communicative situation, such as learning strategies, reflecting on individual situations, and receiving feedback on these reflections, the I-ACE program is an example of a complex intervention. Although previous studies have evaluated the effects of the I-ACE program, participants' experiences with the program have yet to be explored.

The purpose of this study was twofold. First, we aimed to examine the feasibility of providing the Swedish I-ACE program via the Support and Treatment platform. Second, we aimed to explore hearing impaired persons' experiences with the program regarding its content, relevance, manageability, and comprehensibility.

## 2 Materials and methods

### 2.1 Development of the Swedish Individualized Active Communication Education (I-ACE) program

The I-ACE has previously been translated to Swedish and modified to match the changes made for the Swedish version of the ACE program (Öberg et al., 2014a). These modifications included adding a section explaining the audiogram and information on the psychosocial consequences of hearing loss to Chapter 3 (Hagejärd and Teodorescu, 2015). The translated and modified material of the I-ACE program was reviewed and edited to meet the language accessibility guidelines of the 1177.se portal. A team consisting of audiologists with experience using the Swedish ACE program and representatives from the platform worked in close collaboration during the construction of the program. The research team continuously reviewed and edited the material in several rounds to ensure an appropriate distribution of textual content and assignments and interactive material. To further balance the material, and due to the few illustrations of the original I-ACE, an illustrator was involved in creating pictures and animations that were complementary to the textual content.

Once the program was implemented on the platform, three individuals who had previously been fitted with hearing aids at the hearing care clinic in the county of Östergötland were recruited as PPI representatives and assigned the I-ACE. They were asked to provide feedback about the content and relevance of the material to reveal any need for clarification or adjustments of the program. They were also asked to evaluate the manageability and navigation of the program and platform to ensure that the program was functional and manageable for the intended target group. This feedback was provided by completing and submitting a short evaluation form (on paper). The first author (LW) later called the representatives to allow them to elaborate on their answers. This initial feedback did not indicate any difficulties in managing or navigating the program or prompt any necessary changes to the material.

The Swedish I-ACE program consists of five chapters addressing topics such as hearing in background noise, listening to other signals and communication strategies. A more detailed description of the content of the program is presented in Table 1. Each chapter contains written information, reflection tasks and assignments for the participant to complete. Some assignments are intended to be completed when including a significant other or communication partner. These are optional, leaving the participant to decide if and to what extent they want to include someone else in the program.

**Table 1:** Chapters and objectives of the Swedish I-ACE program.

	Title	Objective
Chapter 1	Your listening situation	• To introduce the background and aims of the I-ACE program. • To identify hearing difficulties experienced in everyday life. • To identify communication difficulties and needs that will be addressed in subsequent chapters.
Chapter 2	Communication in background noise	• To use the problem-solving method to manage communication in noise. • To identify and practice different strategies to improve communication in noise. • To use the problem-solving method in different situations in everyday life.
Chapter 3	The ear, hearing and communication around the house	• To gain knowledge of the ear's physiology and to understand the audiogram. • To be able to explain one's own audiogram to family members. • To gain knowledge about strategies to facilitate communication around the house.
Chapter 4	Communication strategies	• To use the problem-solving method when talking to people who are difficult to hear. • To identify and practice communication strategies with people who are difficult to hear. • To use the problem-solving method in different situations in everyday life.
Chapter 5	Listening to other signals	• To use the problem-solving method when listening to other sound sources, such as TVs and telephones. • To gain knowledge of different assistive listening devices. • To use the problem-solving method in different situations in everyday life.

### 2.2 Participants

Participants were recruited using a combination of convenience and purposive sampling. The group-based ACE program is a rehabilitative option at the hearing care clinic in the county of Östergötland. During the pandemic, all group activities were cancelled, resulting in individuals interested in participating in the ACE program being placed on a waiting list. Participants were recruited primarily from this waiting list, and a few were also recruited through other processes at the hearing care clinic and the Swedish Association of Hard of Hearing People (HRF). To ensure that the program was feasible for use in a clinical setting and by a wide range of people, there was an aim to recruit a variety of participants in terms of age, experience with hearing aids, sex, and education level. The inclusion criteria were people with hearing loss who were ≥ 20 years of age with computer experience.

Initially, potential participants were called for a short phone interview, which informed them about the study and assessed eligibility. Those who were eligible and interested in participating were sent written information about the study and informed consent forms. In total, 14 invitations were sent out, and 12 individuals agreed to participate in the study. This sample size is recommended by Julious (2005) as a rule of thumb for pilot and feasibility studies and is a common sample size in qualitative pilot and feasibility studies (O'Cathain et al., 2015).

### 2.3 Procedure

The participants were assigned to the I-ACE program on the Support and Treatment platform. The platform is in a secure section of the 1177.se portal and available only when logged in with e-identification. When the participants first logged on to the platform, they were asked to answer a series of questionnaires to ensure that completing them through the platform worked properly. The questionnaires included the Swedish translations of the Communication and Acceptance Scale (CAS) (Öberg et al., 2021), the Communication Strategies Scale (CSS) (Demorest and Erdman, 1987), the Hearing Handicap Inventory for the Elderly (HHIE) (Ventry and Weinstein, 1982) and the International Outcome Inventory for Hearing Aids (IOI-HA) (Cox et al., 2000). These questionnaires were also answered upon completion of the I-ACE program, including the International Outcome Inventory for Alternative Interventions (IOI-AI) (Noble, 2002). The CAS (score range between 18 and 90) was used to measure the use of communication strategies and acceptance of hearing impairment, with a higher score indicating greater use of communication strategies and acceptance. The CSS (score range between 25 and 125) was used to measure the use of different types of communication strategies, with a higher score indicating greater use of communication strategies. The HHIE (score range between 0 and 100) was used to measure perceived hearing difficulties, with a higher score indicating greater perceived hearing difficulties. The IOI-HA (score range between 7 and 35) was used to measure hearing aid efficacy, with a higher score indicating a better outcome. The IOI-AI (score range between 7 and 35) was used to measure the effects of the intervention, with a higher score indicating a better outcome.

Each week, the participants were assigned a new chapter. At the end of the week, they received written individual feedback on their completed assignments from an audiologist via the platform. Upon completion of each chapter, the participants were asked to answer a few questions, which were the same for all chapters, regarding the content and to provide an estimation of the time required to complete the chapter. After completing the program, the participants were asked to complete an evaluation form regarding their overall experiences in handling and navigating the program and platform.

To gain a better understanding of their experiences with the Swedish I-ACE program, participants were also asked to participate in a semistructured follow-up interview. The interviews were held by the first author (LW) and focused on the general experiences with the program regarding the content, relevance, manageability, and comprehensibility of the material and platform. All participants who had completed at least one chapter of the I-ACE were invited to participate in the follow-up interview. Depending on the participants' preferences, the interview was conducted either by telephone, digitally via Skype or in the clinic.

Ten interviews were carried out. Five interviews were conducted at the clinic, two were conducted digitally, and three were conducted by telephone. Due to technical issues, one of the planned digital interviews was conducted by telephone. The interviews lasted between 30 and 77 min and were transcribed verbatim using NVivo Transcript. The transcripts were then reviewed for accuracy, and any identifying information was removed prior to analysis.

The transcripts were analyzed by the first author (LW) using a version of Framework Analysis. This method is commonly used in the thematic analysis of semistructured interviews (Gale et al., 2013). In this study, a deductive approach was used, with four predetermined topics relevant to the research question: (1) content, (2) relevance, (3) manageability and (4) comprehensibility. These topics were used as the themes in the first draft of the analytical framework. After familiarization with the transcripts, the first author highlighted and subsequently coded phrases relevant to the themes. Similar codes were grouped into categories, sorted according to the suitable theme, and added to the draft of the analytical framework. Using this updated framework, the last author (MÖ) also coded 20 % of the transcripts, which were subsequently compared to those coded by the first author (LW) regarding congruency. Adjustments to the analytical framework were made after discussing discrepancies in coding and the application of the framework. When the analytical framework was established, the data were charted accordingly.

The study was approved by the Swedish Ethical Review Authority (file number 2022-01982-02).

## 3 Results

In total, 12 participants were assigned to attend the Swedish I-ACE program through the Support and Treatment platform. Two participants were excluded because they never started the program (no reason provided). The 10 included participants (females = 7, males = 3) were aged between 46 and 77 years (mean = 62.5 years, SD = 11.98). There was a variety of hearing loss levels, ranging from normal hearing to severe hearing loss. Eight of the participants were hearing aid users with hearing aid experience varying between 2 and 25 years. Two participants were nonusers of hearing aids. Nine participants had a postsecondary education, and one participant had a secondary-level education.

### 3.1 Quantitative results

Eight participants completed all the chapters of the I-ACE program, one participant completed four chapters, and one participant completed three chapters. A more detailed description of the completion rate for each chapter is presented in Table 2. The two participants who did not finish all chapters cited that they found the material repetitive.

**Table 2:** Participant compliance throughout the I-ACE program.

Participant	Chapter 1	Chapter 2	Chapter 3	Chapter 4	Chapter 5
1	✓	✓	✓	✓	✓
2	✓	✓	✓	✕	✕
3	✓	✓	✓	✓	✓
5	✓	✓	✓	✓	✕
7	✓	✓	✓	✓	✓
8	✓	✓	✓	✓	✓
9	✓	✓	✓	✓	✓
10	✓	✓	✓	✓	✓
11	✓	✓	✓	✓	✓
12	✓	✓	✓	✓	✓
Completion rate	100 %	100 %	100 %	90 %	80 %

Note: ✓ = completed chapter, ✕ = did not complete chapter.

Seven participants answered the questionnaires before and after completion of the I-ACE program. Participants tended to increase their CAS and CSS scores and decrease their HHIE score. However, no statistical analyses were performed due to the small sample size. The participants' individual scores are presented in Table 3.

**Table 3:** Individual scores before and after completing the Swedish I-ACE program.

Questionnaire		Participants									
		1	2	3	5	7	8	9	10	11	12
CAS	Pretest	69	51	70	68	57	57	51	61	51	76
	Posttest	77	—	—	—	63	68	65	82	70	74
CSS	Pretest	98	88	91	97	82	97	98	108	85	82
	Posttest	105	—	—	—	100	101	100	117	111	97
HHIE	Pretest	46	64	30	34	62	76	50	28	68	6
	Posttest	42	—	—	—	48	74	58	36	54	4
IOI-HA	Pretest	28	25	28	24	27	31	N/A	N/A	21	34
	Posttest	29	—	—	—	16	29	N/A	N/A	21	34
IOI-AI	Posttest	19	—	—	—	14	25	27	26	17	33

Note: — indicates that the participant did not complete the posttest.

For the CAS, CSS, IOI-HA and IOI-AI a higher score is better. For the HHIE a lower score is better.

Participants 9 and 10 were nonusers of hearing aids and did not complete the IOI-HA.

Eight participants completed the evaluation form after each chapter. Participants found all assignments to be clear, and the distribution of informational content and assignments was deemed balanced. One assignment in Chapters 3 and 5 was deemed ‘too difficult’. In Chapter 3, the assignment of explaining the audiogram was referenced; however, in Chapter 5 the assignment was not specified. In Chapters 4 and 5 some assignments were found to be irrelevant, with participants stating that they were repetitive. Participants reported spending an average of 1.7 h (0.5–5 h) per chapter. Participants generally reported few difficulties in managing and navigating the platform. An overview of the results from the evaluation forms completed after each chapter is presented in Table 4.

**Table 4:** Results of the evaluation forms answered after completing each chapter (*n* = 8).

		Chapter 1	Chapter 2	Chapter 3	Chapter 4	Chapter 5
Were the assignments clear?	Yes	100 %	100 %	100 %	100 %	100 %
	No	0 %	0 %	0 %	0 %	0 %
Were any of the assignments too difficult?	Yes	0 %	0 %	12,5 %	0 %	12,5 %
	No	100 %	100 %	87,5 %	100 %	87,5 %
Were any of the assignments irrelevant?	Yes	0 %	0 %	0 %	25 %	12,5 %
	No	100 %	100 %	100 %	75 %	87,5 %
Was the distribution between reading and assignments well-balanced?	Yes	100 %	100 %	100 %	100 %	100 %
	No	0 %	0 %	0 %	0 %	0 %
Approximately how long did it take to complete this chapter?	Mean	1.5 h	1.7 h	2.25 h	1.6 h	1.25 h
	(Min–Max)	(0.75–2)	(0.5–3)	(0.5–5)	(0.5–4)	(0.75–3.5)

### 3.2 Qualitative results

#### 3.2.1 Evaluation form

Eight participants completed the evaluation form after the program was completed. Participants generally reported few difficulties managing and navigating the platform. The summarized results of the evaluation are presented in Table 5.

**Table 5:** Summarized results from evaluation form, open-ended general questions.

*Did you ever experience difficulties when* log*ging on to the platform?*	None reported.
*Did you ever experience technical difficulties using the platform? If so, please describe in what way.*	None reported.
*How did you find working with the program?*	The majority of participants reported positive experiences while using the program. One participant reported difficulties in finding responses to messages and comments at first.
*Did you ever find it difficult to navigate the program? If so, please describe in what way.*	Over 50 % of participants reported no difficulties in navigating the program. Some participants reported that navigation during the first log in was initially difficult. One participant reported it sometimes being difficult to find the overview while using the program.
*Did you ever find it difficult to know where to write down your answers? If so, please describe when.*	None reported. One participant instead commented feeling that they were sometimes repeating themselves. Another participant commented that some questions consisted of multiple questions in one, making it difficult to answer them all.
*How did you find the distribution of workload among the chapters of the program?*	The majority of participants reported the workload to be generally consistent across chapters. One participant reported finding the workload very unequal but did not elaborate further. Another reported to have deliberately spent more time on a chapter they found especially important.
*Do you have any other comments?*	Several different comments were provided in response to this question. The majority of participants commented that they had found the program to be informative and engaging, contributing to their learning and reflection. Some suggestions for improvements of the material were also reported.

#### 3.2.2 Semistructured interviews

Participants addressed the program in relation to the previously determined themes. The framework was modified, leaving these themes as subthemes for the main theme “program”. The final framework with the main theme, subthemes and categories are presented in Table 6.

(1) *Content*

**Table 6:** Main theme and subthemes with categories of the final framework used in the analysis.

Theme	Subthemes	Categories
Program	Content	• Textual content • Pictures and animations • Suggestions for improvement
	Relevance	• Material • Target group
	Manageability	• Timescale • Flexibility • Platform
	Comprehensibility	• Material • Platform

**Textual content:** The participants generally found the content of the I-ACE to be informative but somewhat repetitive. While most of the participants found parts of the material repetitive, only a few described this as negative:

*“And I think that many questions come up again and again, but I realize that that's the idea, that it should come up again so that it sticks.”*

*– P5*

The level of difficulty of the chapters was described as equal, although many of the participants rated the assignment of describing their own audiogram as more difficult than the other assignments. The same assignment was, nevertheless, the most appreciated among the participants, with one participant stating the following:

*“What has been the absolute best part of this for me is to gain an understanding of the audiogram because I did not have that before.”*

*– P3*

**Pictures and animations:** The participants appreciated the pictures and animations, and some described them as uplifting and clarifying. There was a consensus that the distributions of information, pictures and assignments were balanced:

*“All three parts [text, illustrations and assignments] supported each other well, while the more exploratory questions provided room to deepen or broaden the answers.”*

*– P10*

**Suggestions for improvement:** During the interviews, suggestions to improve the content were provided, ranging from requests to clarify some assignments to the addition of more tips and information to the program.

(2) *Relevance*

**Material:** The content of the chapters and assignments was generally described as relevant. A few participants thought that some assignments after the third chapter were less relevant because they found them repetitive:

*“But, generally speaking, there was a little too much repetition of different situations. I thought it was a bit annoying. I felt that I had already answered this.”*

*– P3*

Target group: Participants generally found the material relevant for the intended target group. Some participants found the material relevant but also expressed that it was not necessarily relevant for themselves. The two participants who did not use hearing aids reported finding the questions and assignments regarding hearing aid use irrelevant. Others stated that depending on how much previous knowledge one had, the relevance could be affected, with one participant stating:

*“For me, this [program] has not added anything. It has not provided any new knowledge for me personally, since I already had all this knowledge before.”*

*– P11*

(3) *Manageability*

**Timescale:** In the interviews, the participants stated that the distribution between written information and assignments was balanced and that the given time frame of one week was sufficient for the scope of each chapter:

“*It was much more manageable, I think, and manageable in terms of the tasks and time and not in any way burdensome. I thought this was fun.”*

*– P8*

Regarding the workload of the chapters, almost all participants reported having to spend more time on chapter three to learn how to interpret their audiogram and later explain it to someone else.

**Flexibility:** Several of the participants stated that the flexibility of being able to work with the chapters based on their own schedule was positive:

*“You can sit down with it in the evening, in the morning, well, at any time really and do this.”*

*– P12*

**Platform:** Participants generally found it easy to use the platform, and none reported difficulties logging on to it. While most participants found the program easy to use and navigate, a few found it difficult to find the messages containing the feedback on their assignments.

(4) *Comprehensibility*

**Material:** Participants reported that the textual information and instructions for the assignments were clear; however, for one of the assignments, one participant found the limits within which they were to answer to be unclear:

*“It was that one [assignment] with the room and the chairs. I understood what was expected, but I was a bit unsure of what kind of freedom I had, what I could move around, how much I could do, so to speak.”*

*– P5*

**Platform:** Some of the participants stated that it was initially difficult to understand how to navigate the platform before they gained some experience working with it. Other participants had prior experience using the platform and reported no difficulties understanding how to use or navigate the platform.

## 4 Discussion

### 4.1 Principal findings

This paper aimed to examine the feasibility of providing the Swedish I-ACE program via the Support and Treatment platform and to explore hearing impaired persons' experiences with the program regarding its content, relevance, manageability, and comprehensibility.

Most of the participants found the content of the I-ACE program to be informative and relevant. During the interviews, the participants emphasized appreciating the assignment of learning to interpret and explain their audiogram the most. Similar results were reported for the Swedish ACE program by Öberg (2017), where several participants commented positively regarding learning more about their hearing loss and audiogram. Interestingly, this assignment, which was deemed the most difficult and took the most time to complete, was the most appreciated.

Participants described parts of the material as repetitive, consistent with the results of previous studies of the ACE program, where some participants suggested “less repetition” to improve the sessions (Hickson et al., 2007; Öberg et al., 2014b). Even though all participants in this study mentioned that the material was repetitive, only a few of them thought of it as negative. Repetition is important when trying to change behavioral patterns, such as changes in the use of communication strategies. Despite the repetitive nature of the ACE and I-ACE programs, both have been shown to increase the use of communication strategies and decrease perceived hearing difficulties (Annerfeldt and Svensson, 2017; Hagejärd and Teodorescu, 2015; Hickson et al., 2007; Öberg, 2017; Öberg et al., 2014a).

When providing an intervention program, it is important that the target group perceives it as relevant. Relevance has been found to be associated with and to influence the motivation to learn and the amount of effort people are prepared to invest (Frymier and Shulman, 1995; Knoster and Goodboy, 2021; Johansen et al., 2023). Perceived relevance has also been found to be a key implication for adherence to interventions (Ellis et al., 2012). The participants who did not complete the I-ACE program were the same ones who found the material less relevant due to repetition.

Regarding the feasibility of providing the program via the Support and Treatment platform, few participants reported difficulties using the platform. These results are consistent with those reported by Malmberg et al. (2023), who used the same platform in their study. Some participants found it initially difficult to understand how to navigate the platform until they gained some experience with it. Participants who had prior experience using the platform reported no difficulties in use or navigation. These results indicate that the platform is easy to use. By providing the I-ACE program via the Support and Treatment platform, a nationally available platform, it is possible to increase accessibility for those who live in rural areas. With the platform being integrated into the public health care system, patients can be assigned the I-ACE program as an intervention complemental to traditional audiologic rehabilitation and receive feedback from health care professionals.

The convenience of providing an individual version of the ACE program via the internet can benefit individuals who might not choose to participate in group sessions for different reasons. When exploring the factors influencing rehabilitation decisions, Laplante-Lévesque et al. (2010) found that the convenience and schedule flexibility of the I-ACE program were beneficial. Similarly, in this study, the participants expressed an appreciation of the flexibility of working with the material according to their own schedule.

Based on the suggestions of improvements provided through the interviews, some changes to the material were made. The content was reviewed, and some formulations were changed to clarify differences in the assignments and to reduce the sense of repetition in the material. Some participants suggested adding more information and tips to facilitate communication. Due to the consensus among participants, it was decided that the scope of the material was appropriate considering the allocated timeframes. However, information regarding different interpretation services available in Sweden, such as written interpretation and sign supported Swedish, was added to the material. This was added since few people are aware that this service is available and can be used as a complement in difficult communication situations. Due to limitations of the platform some of the research teams' requested changes were not possible, such as hiding specific questions that might not be relevant for nonusers of hearing aids.

Although the participants generally found the format of an individual program to be convenient, the request for added tips and information could indicate a need not fully met by an individual program. One benefit of group programs is that they allow participants to share their experiences and learn tips from each other but also provide a group context for individuals who are socially isolated. It is always important to conduct a comprehensive evaluation of which intervention program is better suited for the individual.

### 4.2 Strengths and limitations

There was an intention to recruit a variety of participants in terms of demographic parameters to reflect the wide range of people found in the clinical setting. There was a wide range of ages and experience using hearing aids; however, the sample was predominantly female and well educated. Given that the participants in this study were mainly recruited from the ACE program waiting list, where the majority of individuals were women, it was unsurprising that this was later reflected in the sample. These results are consistent with previous studies of the ACE program (Hickson et al., 2007; Öberg, 2017; Öberg et al., 2014a). The skewed sex distribution is not expected to have greatly affected this study regarding assessing the feasibility of providing the I-ACE program via the Support and Treatment platform, considering that women are more likely to be limited on the internet by the use of technology (Internetstiftelsen, 2022).

Regarding the evaluation of the content and comprehensibility of the I-ACE, it is more likely that the level of education would have affected the results rather than the sex distribution of the participants. Given the strict accessibility guidelines of the platform, the content was reviewed and modified to be easy to read with simplified sentence structures to suit a wide range of educational levels. Recruiting participants from the ACE program waiting list also posed a potential risk of selecting participants with higher motivation and positivity toward the I-ACE program than recruiting participants from the general population. However, it is worth mentioning that the participants who never started or did not complete the I-ACE were all recruited from the ACE program waiting list.

It is important to consider that some of the results of these studies might not be fully generalizable. In Sweden, internet is widely available even in rural areas and 96 % of the population is reported to use the internet. As previously mentioned, a vast majority of the population have used some form of digital health care service (Internetstiftelsen, 2022). This, and the fact that there is a widely available national platform for the public health care system, provides favorable conditions for an online intervention program such as the I-ACE in Sweden. This of course provides a more advantageous starting point for these types of studies. However, in countries where internet availability and use are low, online interventions can be more challenging.

The majority of the qualitative analyses were performed by the first author (LW). Therefore, a few strategies were used to increase the rigor of the analysis. The interview guide was reviewed by the first (LW) and last (MÖ) authors prior to conducting the interviews. Two of the interview transcripts were separately coded and compared for discrepancies before establishing the final framework.

This study represents an initial step in the evaluation of the Swedish I-ACE program provided via the Support and Treatment platform. The results indicate that the content of the I-ACE is informative, relevant, and comprehensible and that it is feasible to provide the program via the online platform. Further research is warranted to determine the effects of the I-ACE program and its implications for clinical settings.

## Funding

This work was supported by The 10.13039/501100009750Kamprad Family Foundation for Entrepreneurship, Research & Charity, Swedish Hearing 10.13039/100005930Research Foundation, The 10.13039/100010799Tysta Skolan Foundation and Grants from Region Östergötland.

## Declaration of competing interest

The authors declare that they have no known competing financial interests or personal relationships that could have appeared to influence the work reported in this paper.

## Figures and Tables

**Fig. 1:**
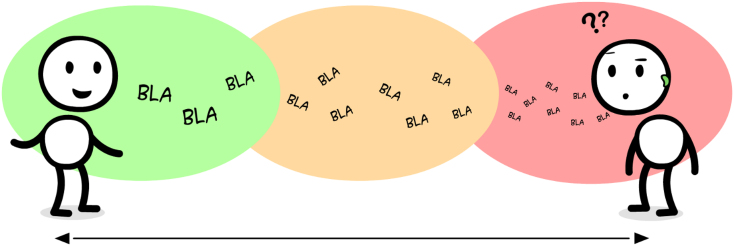
Example of an illustration in the program. Illustrated by Erik Nästesjö Todd.

**Fig. 2:**
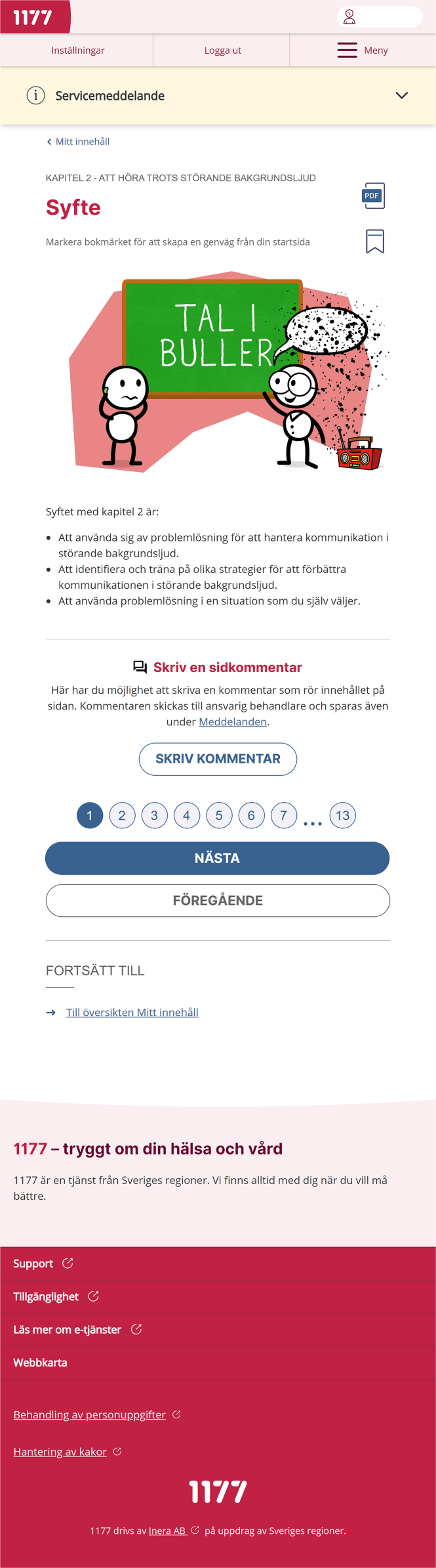
Example of a page in the program on the 1177 Support and Treatment platform. Illustration by Erik Nästesjö Todd.
